# Predicting delayed remission in Cushing’s disease using radiomics models: a multi-center study

**DOI:** 10.3389/fonc.2023.1218897

**Published:** 2024-01-09

**Authors:** Wentai Zhang, Dewei Zhang, Shaocheng Liu, He Wang, Xiaohai Liu, Congxin Dai, Yi Fang, Yanghua Fan, Zhenqing Wei, Ming Feng, Renzhi Wang

**Affiliations:** ^1^ Department of Thoracic Surgery, Peking University First Hospital, Beijing, China; ^2^ Department of Neurosurgery, Chinese Academy of Medical Sciences and Peking Union Medical College, Peking Union Medical College Hospital, Beijing, China; ^3^ Department of Neurosurgery, Jing'an District Center Hospital of Shanghai, Fudan University, Shanghai, China; ^4^ Intensive Care Unit, Beijing Mentougou District Hospital, Beijing, China; ^5^ Department of Neurosurgery, Xuanwu Hospital Capital Medical University, Beijing, China; ^6^ Department of Neurosurgery, Beijing Tongren Hospital, Capital Medical University, Beijing, China; ^7^ Department of Neurosurgery, The Fuzhou General Hospital, Fuzhou, China; ^8^ Department of Neurosurgery, Beijing Tiantan Hospital, Beijing Neurosurgical Institute, Capital Medical University, Beijing, China; ^9^ Department of Neurosurgery, The First Hospital Affiliated to Dalian Medical University, Dalian, China

**Keywords:** Cushing’s disease, radiomics, delayed remission, multicenter study, machine learning

## Abstract

**Purpose:**

No multi-center radiomics models have been built to predict delayed remission (DR) after transsphenoidal surgery (TSS) in Cushing’s disease (CD). The present study aims to build clinical and radiomics models based on data from three centers to predict DR after TSS in CD.

**Methods:**

A total of 122 CD patients from Peking Union Medical College Hospital, Xuanwu Hospital, and Fuzhou General Hospital were enrolled between January 2000 and January 2019. The T1-weighted gadolinium-enhanced MRI images and clinical data were used as inputs to build clinical and radiomics models. The regions of interest (ROI) of MRI images were automatically defined by a deep learning algorithm developed by our team. The area under the curve (AUC) of receiver operating characteristic (ROC) curves was used to evaluate the performance of the models. In total, 10 machine learning algorithms were used to construct models.

**Results:**

The overall DR rate is 44.3% (54/122). According to multivariate Logistic regression analysis, patients with higher BMI and lower postoperative cortisol levels are more likely to achieve a higher rate of delayed remission. Among the 10 models, XGBoost achieved the best performance among all models in both clinical and radiomics models with AUC values of 0.767 and 0.819 respectively. The results from SHAP value and LIME algorithms revealed that postoperative cortisol level (PoC) and BMI were the most important features associated with DR.

**Conclusion:**

Radiomics models can be built as an effective noninvasive method to predict DR and might be useful in assisting neurosurgeons in making therapeutic plans after TSS for CD patients. These results are preliminary and further validation in a larger patient sample is needed.

## 1 Introduction

Cushing’s disease (CD) is a kind of pituitary-dependent Cushing’s syndrome (CS). It is caused by pituitary corticotroph adenoma with a variety of manifestations and complications (1). Its morbidity is ~1.2-2.4 per 100,000 per year (2). CD accounts for around 70% of CS, and the rest are mainly composed of ectopic ACTH syndrome and adrenal tumor or hyperplasia (3). There are plenty of systematic complications of CD due to hypercortisolemia, such as cardiovascular disease, diabetes, and severe osteoporosis (4). The first-line therapeutic plans of CD patients in clinical practice is transsphenoidal surgery (TSS) according to the clinical guideline (5).

According to the latest guideline, immediate remission (IR) was defined as a postoperative cortisol level < 2μg/dL in the first week after TSS (6). If the postoperative cortisol level persistently exceeds the normal range, the surgery was considered a failure. Although IR is often used to evaluate the surgical effects, it is not always consistent with long-term prognosis. There are cases reported by previous studies in which patients with postoperative non-remission achieved remission without additional treatments after long-term follow-up, which is defined as delayed remission (DR) (7).

Adjuvant therapies are usually necessary in CD patients with DR. Therefore, it’s important for doctors to verify DR. A previous study used a traditional biostatistical method to detect risk factors of DR, and the result showed that gender, postoperative 24h UFC, and pathological results are significantly related to DR (7). Nevertheless, the prognosis of CD should not be determined by a single factor. According to a previous study by our team, five machine learning (ML) based models incorporating only clinical data were trained. The highest area under the curve (AUC) value of the receiver operator characteristic curve (ROC) was 0.762 in adaboost (8). MRI data were not available in our previous study which is conducive to the prediction of DR.

ML is a branch of artificial intelligence that can learn knowledge by extracting patterns from databases automatically (9). ML has been used in several studies in the prediction of prognoses and the radiotherapeutic response of pituitary adenoma (8, 10, 11). ML models produce better accuracy and discrimination ability for classification tasks compared with traditional biostatistical methods. Radiomics method is an emerging technology based on ML and it can extract data from imaging that reflects biological information of the focus (12). Compared with the traditional method of incorporating imaging information, radiomics has two main advantages. First, it enables the automatic extraction of radiomics features. Second, high-dimensional radiomics is conducive to identifying the heterogeneity within the regions of interest (ROIs) and exploring the spatial complexity of the disease (13). Thus, in the present study, we used radiomics method and ML incorporating clinical features and MRI data from three clinical centers as input to build models to predict DR.

## 2 Methods

### 2.1 Study population

A total of 122 participants with CD were enrolled in this study at the department of neurosurgery in Peking Union Medical College Hospital (PUMCH), Xuanwu Hospital (XWH), and Fuzhou General Hospital (FGH) between January 2000 and January 2019. Inclusion criteria of participants are: 1) the clinical manifestations of hypercortisolemia (14); 2) positive result of pituitary tumors on pre-operative T1 weighted gadolinium-enhanced MRI; 3) meeting the endocrine diagnostic criteria described in the section “Diagnosis of Cushing’s Disease”; 4) not meeting the criteria for IR; 5) MRI images available. The present study was approved by the local ethical review committee of PUMCH, XWH, and FGH. Informed consents of all participants were obtained.

Participants were categorized into the DR group or the non-DR group, according to the long-term outcome. DR refers to those patients diagnosed as non-remission in the first week after TSS and remission after at least one year without adjuvant therapies. Participants who did not meet the criteria for DR in one-year follow-up were assigned to the non-remission group.

### 2.2 The diagnosis of Cushing’s disease

All participants routinely underwent T1-weighted, T2-weighted, T1-weighted Gadolinium-Enhanced (T1-GE), or Dynamic Gadolinium-Enhanced T1-weighted (DGE-T1) MRI. The diagnosis of pituitary adenoma was suspected if there is a relatively hypointense region in the pituitary gland using T1-GE MRI or DGE-T1 MRI when T1-GE MRI failed. In addition, even though Bilateral petrosal sinus sampling (BIPSS) is key for the confirmation of Cushing’s disease, but it’s not routinely performed due to medical insurance policy and financial reasons in China. Instead, combined low-dose and high-dose dexamethasone suppression tests (LHDDST) were routinely administered. In LDDST, a dose of 0.5mg dexamethasone was administered to patients every 6 hours for two days. If 24-hour urinary free cortisol (UFC) is lower than 12.3ug/d on the second day or plasma cortisol level lower than 1.8ug/dL in the morning of the third day, cortisol was thought to be suppressed. In HDDST, 2 mg dexamethasone was administered every 6 hours for two days. Cortisol was considered suppressed if it was reduced by more than 50% compared to its original level. The failure of LDDST and the success of HDDST indicate the diagnosis of CD.

In some cases where the tumor outline was vague in MRI, bilateral petrosal sinus sampling (BIPSS) with desmopressin stimulation test was used. In general, 10 mg desmopressin was given to participants to stimulate ACTH. In the circumstances that the ratio of ACTH concentration in the inferior petrosal sinus to that in the peripheral vein is larger than 2 in basal state or larger than 3 after desmopressin stimulation, the diagnosis of CD could be made.

The final diagnosis of CD was based on the combined evidence, including MRI, clinical manifestations, combined LDDST, and HDDST and BIPSS with desmopressin stimulation. All surgeries were operated by experienced pituitary neurosurgeons who perform more than 50 saddle region surgeries per year.

### 2.3 Postoperative management and clinical data

After TSS, endocrinological tests were performed to detect immediate remission for 7 consecutive days. Hormone replacement therapy was initiated immediately if postoperative morning plasma cortisol was lower than 5μg/dL (138nmol/L) (3). Patients were examined in 1, 3, and 12 months after TSS and continued re-examination every year.

In total, 18 clinical features were selected, including gender, age, BMI, disease duration, tumor size, Knosp grade, preoperative plasma cortisol level (PrC), preoperative ACTH (PrACTH), preoperative 24-h UFC, combined LDDST and HDDST, pathological confirmation, cerebrospinal fluid leakage (CSF leakage), cavernous sinus invasion (CSI), MRI, ki-67, PoC, postoperative ACTH, and postoperative 24-h UFC. The disease duration was defined as the interval between the symptom onset and the attendance. Macroadenoma was defined as the tumor whose diameter was ≥1cm, and microadenoma was defined as the tumor whose diameter was <1cm. CSI and CSF leakage were evaluated by surgeons intraoperatively (15). CSI positive was defined as the existence of intraoperative cavernous sinus wall defect. Pathological results were collected according to postoperative pathology and immunohistochemical staining. The Ki-67 level was defined as high (the index ≥3%), or low (<3%) (16).

### 2.4 Extracting radiomics features using T1-weighted gadolinium-enhanced MRI images

We first segmented the three-dimensional ROIs delineating the tumors on T1-weighted gadolinium-enhanced MRI images using an automatic tumor masking method developed by our team (17). To ensure the quality of the tumor segmentation, the automatic segmentations were further manually modified by two experienced radiologists (with more than 6 years of neuroimaging experience). We then extracted a total of 1197 radiomics features using a typical radiomics features extraction method – PyRadiomics (https://github.com/Radiomics/pyradiomics). Continuous variables were normalized using z-scores. Using the recursive feature elimination (RFE) algorithm with 5-fold cross-validation, radiomics features with high representation ability were selected for the next step of constructing radiomics models.

### 2.5 Constructing the clinical models and radiomics models

We constructed clinical and radiomics models based on clinical features (18 in total) and radiomics features (22 in total), respectively. To thoroughly exploit the intrinsic relationships of the data and compare the representation differences between the models, we employed and evaluated the accuracy and AUC values of 10 machine learning models, including the ElasticNet (18), the Linear Support Vector Classifier (Linear SVC), Random Forest Classifier (RF) (19), Extra Trees Classifier (ET) (20), K Neighbors Classifier (KNN), Decision Tree Classifier (DT) (21), Gradient Boosting Classifier (GDBT) (22), Adaptive Boosting Classifier (AdaBoost) (23), Multi-layer Perceptron (MLP) (24), and Extreme Gradient Boosting Classifier (XGBoost) (25). The five-fold cross-validation method was performed instead of splitting the patients into a training and a test group during the process of model construction.

### 2.6 Model interpretation

To evaluate the contribution of each feature in our participants, we employed 2 algorithms: 1) the SHAP (SHapley Additive exPlanations) algorithm, which calculates the marginal contribution of each feature in the study population (26); 2) the LIME (Local Interpretable Model-Agnostic Explanations) algorithm, which can calculate the approximate expression of the constructed model and explain the contribution of each feature to specific patients (27).

### 2.7 Statistical analysis

All statistical analyses were performed with IBM SPSS Statistics 23.0 software (IBM Corporation) and RStudio software (1.2.5042). Statistics were defined as significant if P<0.05, two-sided. Continuous variables were presented either with mean ± standard deviation (with normal distribution) or median and interquartile (IQR)(with non-normal distribution). Wilcoxon test was used for the comparison of non-normal continuous variables. Categorical variables were presented as the frequency and the percentage, and analyzed with the chi-square test or Fisher’s exact test. Univariate logistic regression analysis was used to determine the risk factor for DR. Variables with P <0.05 were then used to determine the independent risk factor in multivariate LR. Receiver Operating Characteristic (ROC) curves were used to determine the cut-off point of continuous variables.

## 3 Results

### 3.1 The characteristics of participants

**Figure 1:**
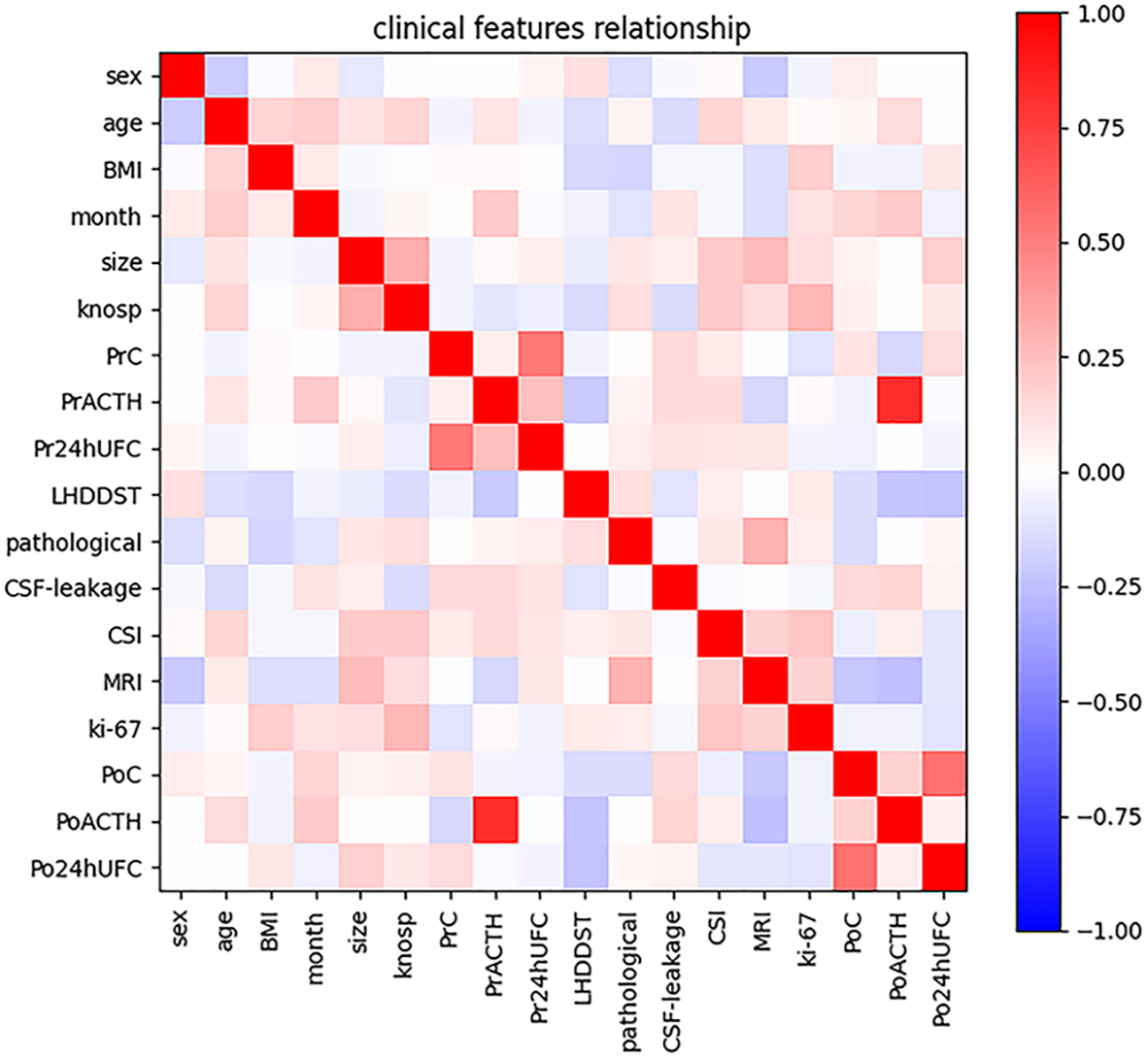
Intensity of correlations among clinical variables. The color bar in the right side represents the intensity of the correlations.

The characteristics of the participants are summarized in **Table 1**. In total, 122 participants met the inclusion criteria mentioned above and were included in the final analysis. And the rate of DR is 44.3% (54/122). All participants were suggested to be reexamined in 1, 3, 6, and 12 months after surgery. In total, 54 participants got DR. Ten participants went into remission in 1 month after surgery, 16 in 3 months, 24 in 6 months, and 4 in 12 months. To confirm the consistency of data from three clinical centers, we examined the correlation of all patients’ clinical information. And several characteristics with strong correlation can be obtained: 1. Knosp grade is positively correlated with tumor diameter. 2. urinary cortisol was positively correlated with blood cortisol both preoperatively and postoperatively. 3. PoACTH is positively correlated with PrACTH (**Figure 1**).

**Table 1:** The clinical characteristics of participants.

Characteristic	Delayed Remission	Non-remission	P-value
**Gender** Male Female	54 10 44	68 15 53	0.630
**Age**	31.50 (26.25-43)	28 (22.75-38)	0.069
**BMI**	26.71 (25.14-29.14)	25.48 (23.72-28.09)	0.014
**Disease duration**	49 (21.75-84)	43 (18-74.75)	0.802
Tumor size			
Microadenoma Macroadenoma	35 19	53 15	0.108
Knosp grade			
0-II III-IV	48 6	65 3	0.182
**PrC**	26.90 (23.49-33.58)	26.41 (23.22-33.78)	0.742
**PrACTH**	91.25 (58.3-144.5)	78 (53.55-103.5)	0.087
**Pr24h-UFC**	585.3 (309.66-1026.316)	525.99 (361.08-754.25)	0.307
LHDDST			
Positive Negative	41 13	58 10	0.189
Pathology			
Positive Negative	47 7	53 15	0.194
CSF-leakage			
Positive Negative	9 45	12 56	0.887
CSI			
Positive Negative	6 48	7 61	0.885
MRI			
Positive Negative	49 5	49 19	0.010
Ki-67			
≥3% <3%	15 39	12 56	0.181
**PoC**	12.24 (8.12-19.18)	18.59 (10.86-25.65)	0.004
**PoACTH**	36.17 (23.45-56.90)	40.63 (33.99-55.98)	0.050
**Po24h-UFC**	244.99 (140.52-605.98)	478.28 (104.31-1003.72)	0.314

BMI, body mass index; PrC, preoperative morning serum cortisol; PrACTH, preoperative morning ACTH level; Pr24hUFC, preoperative 24-hour urine free cortisol; LHDDST, low-dose and high-dose dexamethasone suppression test; CSF, cerebrospinal fluid; CSI, cavernous sinus invasion; PoC, postoperative immediate morning serum cortisol; PoACTH, postoperative immediate morning ACTH level; Po24hUFC, postoperative immediate 24-hour urine free cortisol.

### 3.2 Univariate and multivariate LR analysis

Univariate and multivariate LR analyses were also used to determine the independent risk factors for DR. According to univariate analysis (**Table 2**), patients with older age, higher BMI value, positive MRI findings of tumors, and lower PoC were more likely to achieve DR. According to multivariate LR analysis (**Table 2**), patients with higher BMI were more likely to achieve DR. Patients with lower PoC were linked to a higher chance of DR.

**Table 2:** Univariate and multivariate LR analysis for the determination of independent risk factors for DR.

Variable	Univariate LR analysis			Multivariate LR analysis		
	OR	95% CI	p-value	OR	95% CI	p-value
**Gender**	0.803	0.328-1.964	0.631			
**Age**	1.032	1.001-1.063	**0.046**	1.031	0.997-1.066	0.07
**BMI**	1.136	1.024-1.261	**0.016**	1.132	1.009-1.270	**0.035**
**Disease duration**	1.000	0.992-1.007	0.899			
**Tumor size**	1.909	0.857-4.251	0.114			
**Knosp grade**	2.708	0.645-11.377	0.174			
**PrC**	1.001	0.968-1.036	0.949			
**PrACTH**	1.001	0.998-1.005	0.467			
**Pr24hUFC**	1.000	1.000-1.001	0.203			
**LHDDST**	0.544	0.218-1.359	0.193			
**Pathology**	1.900	0.714-5.060	0.199			
**CSF leakage**	0.933	0.361-2.411	0.887			
**CSI**	1.089	0.343-3.454	0.885			
**MRI**	3.800	1.314-10.987	**0.014**	2.842	0.846-9.547	0.091
**Ki-67**	1.795	0.758-4.251	0.184			
**PoC**	0.963	0.932-0.995	**0.024**	0.962	0.929-0.998	**0.037**
**PoACTH**	0.994	0.983-1.005	0.299			
**Po24hUFC**	1.000	1.000-1.000	0.839			

BMI, body mass index; PrC, preoperative morning serum cortisol; PrACTH, preoperative morning ACTH level; Pr24hUFC, preoperative 24-hour urine free cortisol; LHDDST, low-dose and high-dose dexamethasone suppression test; CSF, cerebrospinal fluid; CSI, cavernous sinus invasion; PoC, postoperative immediate morning serum cortisol; PoACTH, postoperative immediate morning ACTH level; Po24hUFC, postoperative immediate 24-hour urine free cortisol. All bold values are less than 0.05.

### 3.3 ROC curve analysis

**Figure 2:**
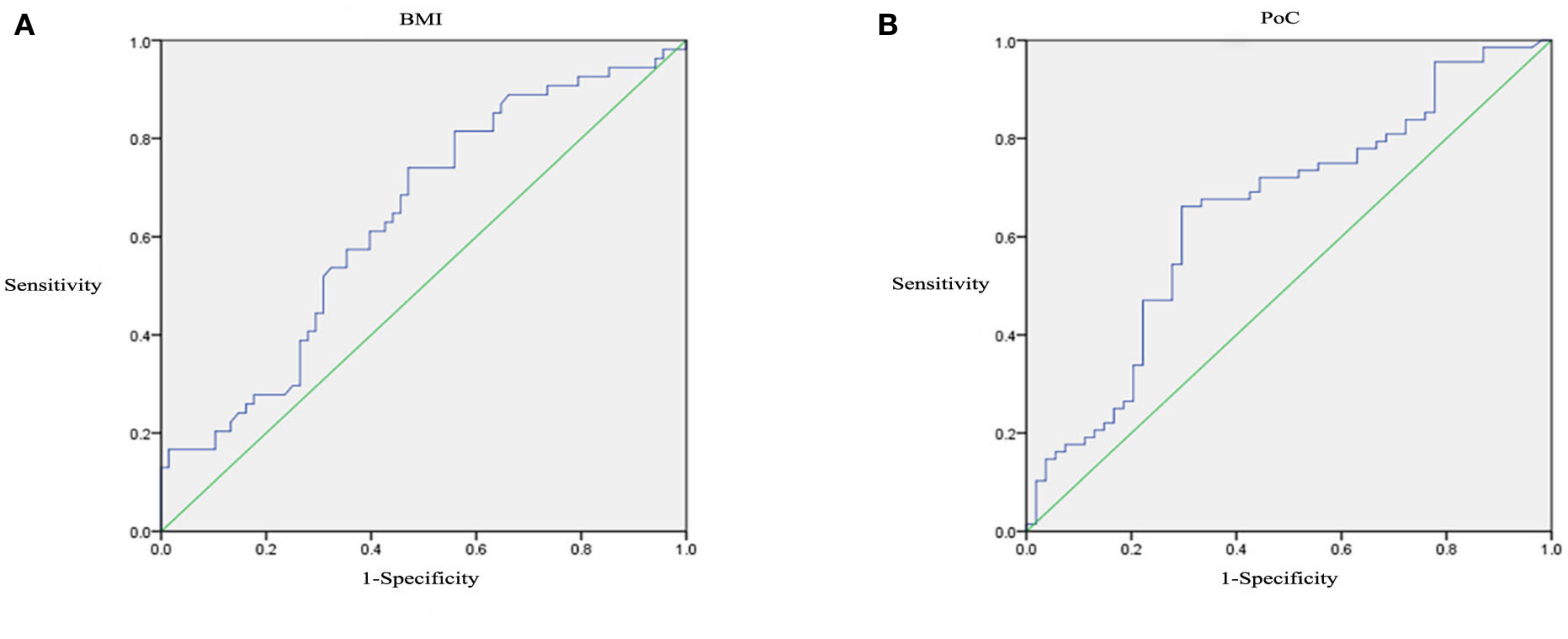
Receiver operating characteristic (ROC) curves analysis of BMI **(A)** and PoC **(B)**.

Receiver operating characteristic curves analysis was performed to detect the optimal thresholds of clinical features. At the cutoff of 25.58, BMI predicted DR with a sensitivity of 0.741 and a specificity of 0.529 (AUC=0.637, P=0.01, 95% CI: 0.538-0.736; **Figure 2A**). At the cutoff of 15.68, PoC predicted DR with a sensitivity of 0.662 and a specificity of 0.704 (AUC=0.651, P=0.004, 95% CI: 0.552-0.750; **Figure 2B**).

### 3.4 Predictive performance of clinical models and radiomic models

**Figure 3:**
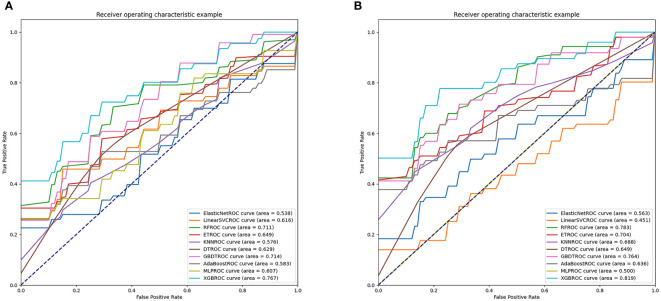
AUC values of clinical models and radiomics models. **(A)** shows the performances of clinical models. **(B)** shows the performances of radiomics models.

When all 18 clinical features were incorporated, the best performance was achieved in XGBoost (AUC=0.767) in the test dataset which outperformed GBDT (AUC=0.714), RF (AUC=0.711), ET (AUC=0.649), DT (AUC=0.629), Linear SVC (AUC=0.616), MLP (AUC=0.607), AdaBoost (AUC=0.583), KNN (AUC=0.576) and Elastic Net (AUC=0.538) (**Figure 3A**). Through the feature selection procedure, a total of 22 radiomics features were selected. A total of 40 features were obtained including clinical and radiomics features. Then the F test analysis of variance was used to further select features. Finally, 31 clinical and radiomics features were used to train clinical-radiomics models. **Figure 3B** shows the performance of different models. The best performance was observed in XGBoost (AUC=0.819) which was followed by RF (AUC=0.783), GBDT (AUC=0.764), ET (AUC=0.704), KNN (AUC=0.688), DT (AUC=0.649), AdaBoost (AUC=0.636), Elastic Net (AUC=0.563), MLP (AUC=0.500) and Linear SVC (AUC=0.451). The performances of clinical models and radiomics models are shown in **Table 3**.

**Table 3:** The performances of clinical models and radiomics models.

Algorithms	Clinical Models		Radiomics Models	
	ACC	AUC	ACC	AUC
**ElasticNet**	0.604	0.538	0.546	0.563
**LinearSVC**	0.613	0.616	0.533	0.451
**RF**	0.607	0.711	0.679	0.783
**ET**	0.549	0.649	0.606	0.704
**KNN**	0.531	0.576	0.614	0.688
**DT**	0.631	0.629	0.623	0.649
**GBDT**	0.624	0.714	0.605	0.764
**AdaBoost**	0.589	0.583	0.604	0.636
**MLP**	0.541	0.607	0.510	0.500
**XGBoost**	0.681	0.767	0.695	0.819
**MAX**	0.681	**0.767**	0.695	**0.819**

Acc, accuracy; AdaBoost, Adaptive Boosting Classifier; AUC, area under the curve; DT, Decision Tree Classifier; ET, Extra Trees Classifier; GDBT, Gradient Boosting Classifier; KNN, K Neighbors Classifier; Linear SVC, Linear Support Vector Classifier; MLP; Multi-layer Perceptron; RF, Random Forest Classifier. All bold values are less than 0.05.

### 3.5 The importance of clinical features

**Figure 4:**
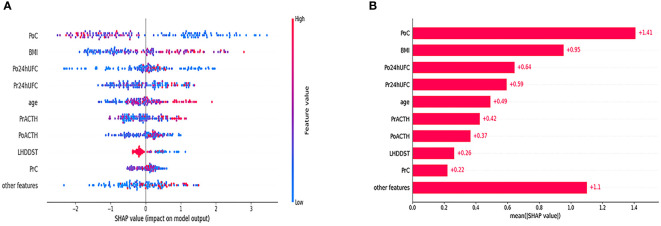
Density scatter was plotted in **(A)**. The bar in the right side of **(A)** shows the feature value. The redder the color is, the larger the feature value is. The bluer the color is, the smaller the feature value is. The minus value of SHAP value tends to indicate a lower possibility of DR. The positive value of SHAP value tends to indicate a higher possibility of DR. **(B)** Shows the order of the importance of SHAP value.

The SHAP results showed that the top 2 features were PoC and BMI, consistent with the result of multivariate LR analysis. We plotted a density scatter plot and bar chart to show the SHAP value in **Figure 4**. Other features remained lower in mean SHAP values.

**Figure 5:**
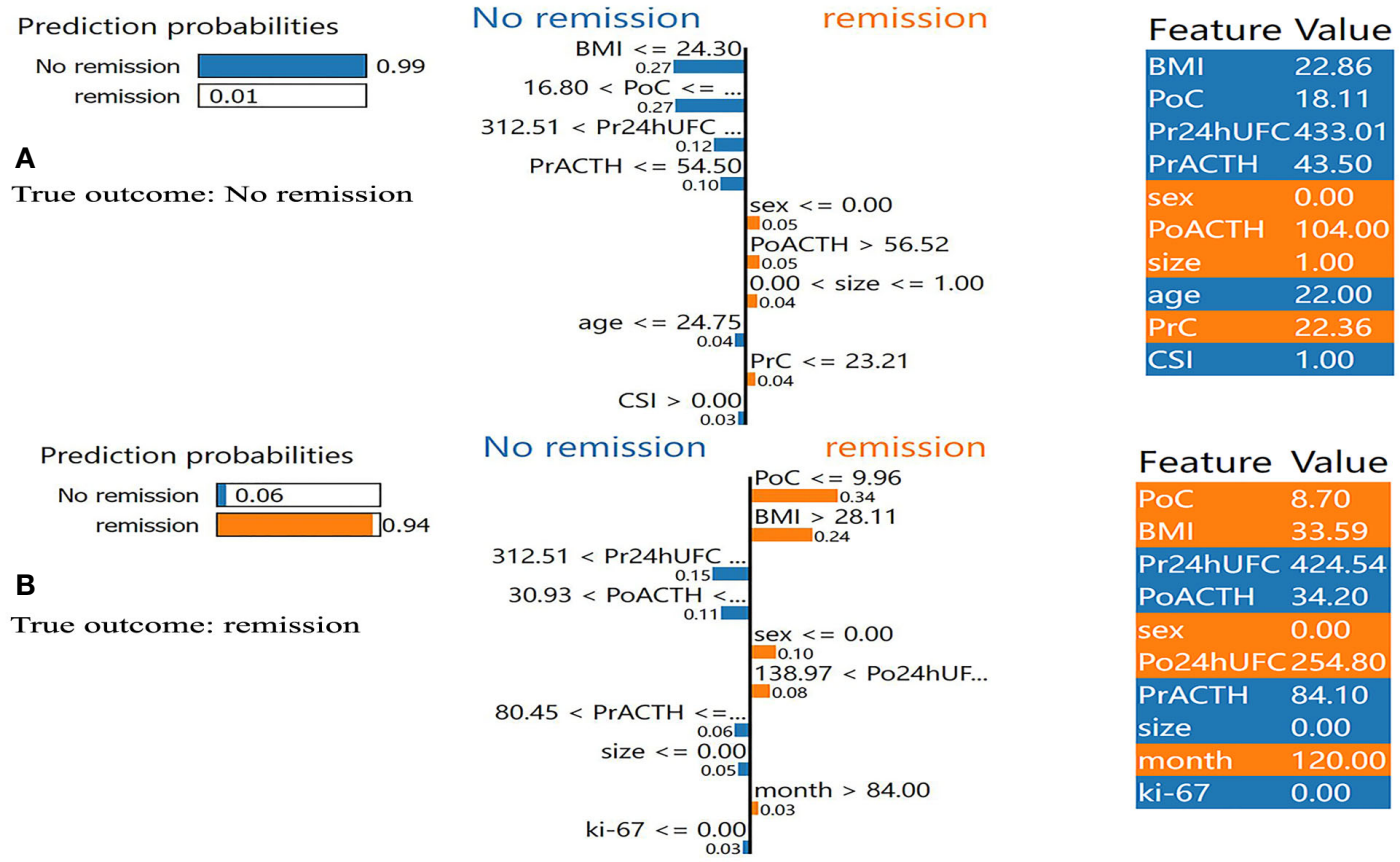
Results of local interpretable model-agnostic explanation (LIME) with XGBoost algorithm. The figure reveals the role of each feature contributing to the probability of DR. The first column shows the probability output by XGBoost. The second column shows the contributions of the probability of each feature. The value below the feature shows the weight coefficient by LIME. The third column shows the original value of the features. **(A)** Shows true negative. **(B)** Shows true positive. BMI, body mass index; PrC, preoperative morning serum cortisol; PrACTH, preoperative morning ACTH level; Pr24hUFC, preoperative 24-hour urine free cortisol; CSI, cavernous sinus invasion; PoC, postoperative immediate morning serum cortisol; PoACTH, postoperative immediate morning ACTH level; Po24hUFC, postoperative immediate 24-hour urine free cortisol.

**Figure 6:**
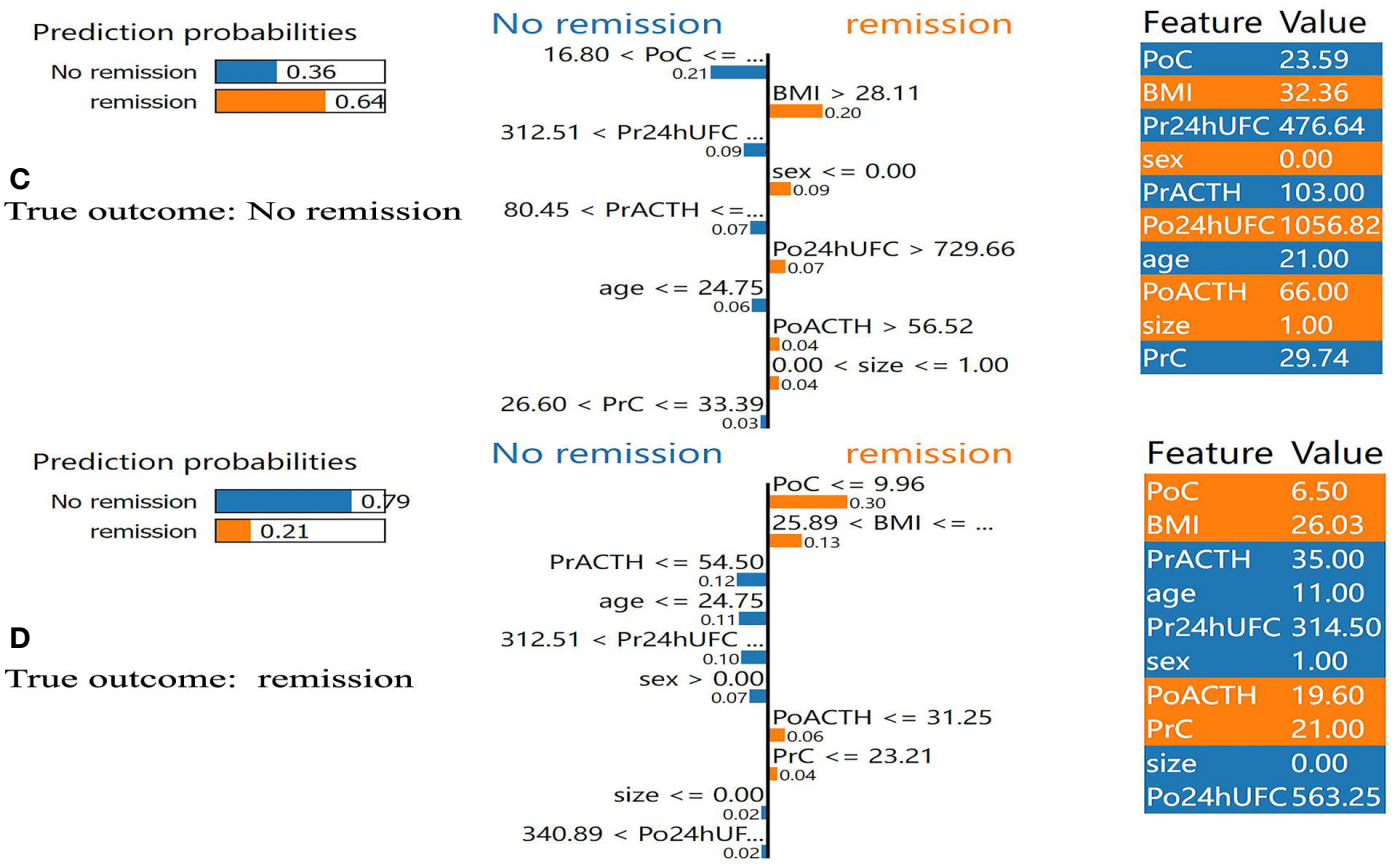
Results of local interpretable model-agnostic explanation (LIME) with XGBoost algorithm. The figure reveals the role of each feature contributing to the probability of DR. The first column shows the probability output by XGBoost. The second column shows the contributions of the probability of each feature. The value below the feature shows the weight coefficient by LIME. The third column shows the original value of the features. **(C)** Shows false positive. **(D)** Shows false negative. BMI, body mass index; PrC, preoperative morning serum cortisol; PrACTH, preoperative morning ACTH level; Pr24hUFC, preoperative 24-hour urine free cortisol; CSI, cavernous sinus invasion; PoC, postoperative immediate morning serum cortisol; PoACTH, postoperative immediate morning ACTH level; Po24hUFC, postoperative immediate 24-hour urine free cortisol.

In addition, we found that several key clinical features stand out when using LIME algorithm (explained specific predictions for one patient by learning an interpretable model). 4 exemplary patients were further described here. Patients A and B were correctly predicted by our final model (**Figure 5**): patient A was predicted as having a 99% probability of no remission, which was mainly based on the relatively low value of BMI (BMI=22.86), the high value of PoC (PoC=18.11μg/dL), the high value of Pr24h-UFC (433.01μg/24h), and relatively low value of PrACTH (43.5pg/ml); Patient B was predicted as having a 94% probability of DR, which was mainly due to a low PoC value (PoC=8.70μg/dL) and high BMI (BMI=33.59). P:atients C and D were incorrectly predicted by the final model (**Figure 6**): patient C was incorrectly predicted as having a 64% probability of DR, mainly due to a relatively high BMI (32.36), female gender, and high Po24h-UFC (1056.82μg/24h); patient D was incorrectly predicted with a 79% probability of no remission, mainly based on a relatively low PoC (6.5μg/dL) and relatively high BMI (26.03).

## 4 Discussion

In the present study, we developed 10 ML-based models to predict the DR of patients with CD. The AUC values range between 0.538 and 0.767 in clinical feature models, 0.500 and 0.819 in radiomic models. We found that the XGBoost model provided the best performance (AUC=0.819) when clinical features and radiomic features were both incorporated to predict the DR of CD patients. XGBoost radiomic model performed conspicuously better than BMI and PoC, respectively. Therefore, XGBoost radiomic model was chosen to be the final model of the present study.

According to the current guidelines, IR within 1 week is used to determine the response to surgeries (5). However, previous studies have revealed that long-term postoperative cortisol may be lower than the level measured within one week after surgery (28, 29). Patients with postoperative hypercortisolism often need adjuvant therapies (30). The remission rate of repeated surgery is conspicuously lower than the first operation, and the incidence of complications is also higher in repeated surgery (31). Therefore, neurosurgeons need to recognize patients with DR correctly.

There are two main hypotheses about the mechanism of DR in CD. First, in patients with CD, adrenal hyperplasia may cause hypercortisolemia, and gradually disappear after TSS (7). Second, the TSS destructs the blood supply of residual tumor cells and causes the necrosis of ACTH- secreting tumor cells. Therefore, the cortisol level gradually decreased to cause DR without adjuvant therapies (7, 32–34). Valassi et al. indicated that patients’ cortisol levels should be closely monitored to avoid unnecessary adjuvant therapies or repeated surgeries. Therefore, we developed ML-based radiomic models to identify patients possibly associated with postoperative DR to facilitate long-term follow-up and treatment strategies.

Previous studies about DR of patients with CD were mainly about the retrospective analysis of risk factors. However, it is generally believed that a single factor should not determine the prognosis of CD, but combined factors (35). By far, there have been many studies demonstrating that ML-based models could be used to predict the prognosis of saddle region disease and other tumors (36–38). Thus, our team developed ML-based models incorporating clinical features to predict DR with the highest AUC value of 0.762 in the test dataset (8). Our previous study had some limitations. First, it was a single-center study without external validation. Second, patients with postoperative non-remission who achieved remission after adjuvant therapies were excluded from the study, and they were likely to maintain non-remission if they were not treated with adjuvant therapies.

Some ML-based models were criticized for the lack of transparent learning and the outputs, known as the “black box”. The excellent performance of the models is not enough for its clinical application. Interpretability is even more important for doctors. Thus, in the present study, we used the SHAP value for model interpretation. SHAP value is often used in the analysis of the effect of each feature contributing to the model. The SHAP value explains the effect of a particular feature at a particular value by comparing it with the prediction of a feature at a baseline value. According to SHAP value, PoC and BMI were the top 2 most important factors contributing to the model (**Figure 4**). LIME algorithm was also used to exploit the impact of features further. The LIME algorithm can explain a complex model’s prediction by simulating an interpretable model around the original input with local fidelity. In the present study, LIME algorithm visualized the interpretable process of the model on specific 4 patients.

There are advantages of the present study. First, this is a radiomic study which makes it more comprehensive and accurate than our previous one (8). Second, this is a multicenter study which gives the model higher robustness. There are also disadvantages to the study. First, though it is a multicenter study, the study’s sample size is relatively small due to the difficulty of obtaining the MRI, which made it vulnerable to overfitting when modeling. Second, though a one-year follow-up was performed to distinguish patients with DR, long-term follow-up is still necessary for future studies. Third, only patients with visible pituitary adenoma on MRI were included which represent only a fraction of patients affected by this condition.

## 5 Conclusions

In the present study, we developed clinical and radiomics models that performed well in predicting DR after TSS and XGBoost radiomics model performed the best. We suggest that patients with CD who failed to achieve immediate remission should not be treated with adjuvant therapies immediately after TSS. Instead, the probability of DR should be predicted by our radiomics model to formulate postoperative treatment plans. However, these results are preliminary and further validation in a larger patient sample is needed.

## Data availability statement

The data analyzed in this study is subject to the following licenses/restrictions: The datasets can be available according to reasonable requests. Requests to access these datasets should be directed to WZ, zhangwt2020@163.com.

## Ethics statement

The studies involving humans were approved by local ethical review committee of Peking Union Medical College Hospital, Xuanwu Hospital, and Fuzhou General Hospital. The studies were conducted in accordance with the local legislation and institutional requirements. Written informed consent for participation was not required from the participants or the participants’ legal guardians/next of kin in accordance with the national legislation and institutional requirements.

## Author contributions

WZ, HW, XL and CD contributed equally to the present study. Each author contributes to the article in data collecting and analysis. MF, RW, and ZW take final responsibility for this article. All authors contributed to the article and approved the submitted version.
